# Dosimetric Comparison of Whole Breast Radiotherapy Using Field-in-Field and Volumetric Modulated Arc Therapy Techniques in Left-Sided Breast Cancer Patients

**DOI:** 10.7759/cureus.15732

**Published:** 2021-06-18

**Authors:** Asmara Waheed, Sumera Butt, Ali Ishtiaq, Muhammad Atif Mansha, Sana Mehreen, Mohsin Raza, Muhammad Yousaf

**Affiliations:** 1 Department of Clinical and Radiation Oncology, Shaukat Khanum Memorial Cancer Hospital and Research Centre, Lahore, PAK; 2 Department of Clinical and Radiation Oncology, Shaukat Khanum Memorial Cancer Hospital and Research Center, Lahore, PAK; 3 Department of Clinical and Radiation Oncology, Shaukat Khanum Memorial Cancer Hospital & Research Centre, Lahore, PAK

**Keywords:** whole breast radiotherapy, field in field, volumetric modulated arc therapy, organs at risk, conformity index

## Abstract

Introduction

The radiotherapy of left-sided breast cancers is challenging because of neighboring critical organs, posing an increased risk of complications. Various radiation delivery techniques have been used to deliver the desired dose of radiation to the target area while keeping the doses to nearby structures within constraints.

The main aim of this study is to quantify doses delivered to the organs at risk (OARs) including heart, left lung, spinal cord, and contralateral breast, and to the planning target volume (PTV) using Field-in-Field (FIF) and Volumetric Modulated Arc Therapy (VMAT).

Patients and methods

A retrospective review of 15 left-sided breast cancer patients was done. All the patients underwent breast-conserving surgery and adjuvant radiation. For every patient, two different radiation treatment plans were formulated and compared for the PTV coverage and doses to OARs, including heart, ipsilateral lung, spinal cord, and contralateral breast.

The radiation treatment techniques utilized for this purpose were FIF and VMAT. The homogeneity index (HI), and conformity index (CI) required for the treatment planning were also calculated. Data was analyzed using Statistical Package for the Social Sciences (IBM Corp., Armonk, USA). An Independent T-test was used for statistical analysis.

Results

The mean age was 41 years and the majority of them were stage II. Total nine patients were given 4005centi Gray (cGy) in 15 fractions (fr) followed by 10Gy boost, hence receiving a total dose of 5005cGy in 20fr. While remaining six patients were given a total dose 4005cGy in 15fr without any boost.

All patients were hypofractionated and the dose was delivered at a rate of 267cGy per fr. The FIF technique utilized in breast cancer radiation significantly reduced the mean doses to OARs: mean heart dose (3.81cGy), ipsilateral lung dose (V16- 15cGy), mean contralateral breast dose (0.03cGy), and maximum spinal cord dose (0.18cGy); as compared to VMAT technique which delivered comparatively higher doses: mean heart dose (8.85cGy), ipsilateral lung dose (V16- 19.82cGy), mean contralateral breast dose (4.59cGy), and maximum spinal cord dose (7.14cGy).

There was a significant mean difference between doses of OARs and all p-values were statistically significant (p<0.005). Moreover, the FIF technique also improves the dose distribution of PTV in terms of dose homogeneity. However, the conformity index is more enhanced with VMAT as opposed to FIF.

Conclusion

The FIF technique is more advantageous than the VMAT planning technique because it provides better dose distribution in terms of PTV coverage and significantly lower doses to OARs in radiotherapy to left-sided breast cancer.

## Introduction

Breast cancer is the most common cancer in females. According to Globocan statistics 2018, the worldwide burden of breast cancer is 11.6% [1]. The most common approach to treat breast cancers is breast-conserving surgery (BCS) followed by postoperative external beam radiation therapy (RT) [2]. With the late presentation of breast cancers in underdeveloped countries, mastectomy followed by adjuvant radiation to the chest wall is the most frequently used treatment strategy [3]. An overview of randomized trials in early breast cancer patients has shown the effect of adjuvant radiation therapy after breast-conserving surgery in the form of improved local control and overall survival [4].

Traditionally, adjuvant radiation therapy to the breast was delivered with conventional tangential beams which include part of the anterior thoracic cavity, consequently increasing the doses to organs at risk (OARs), in particular heart and ipsilateral lung. These approaches delivered homogenous doses to the entire volume including OARs resulting in considerable normal tissue toxicity. Furthermore, It is also proven that breast cancer patients treated on the left side have a moderately increased risk of cardiac toxicity and morbidity [5]. Therefore, the deleterious effects of radiation therapy especially cardiotoxicity, not only have increased the risk of death but also hinder determining the actual survival benefit [6].

In parallel, the advancements in external beam radiation treatment have revolutionized the delivery techniques to the whole breast and have improved radiation dose conformity, thereby limiting exposure to healthy tissue. As compared to conventional tangential fields, the computed tomography (CT)-based conformal tangential radiotherapy (CRT) in whole breast RT has significantly spared the doses to the OARs [7]. Although, dose distribution obtained from conventional tangential beams has been improved over the years with the use of beam modifiers like wedge filters particularly enhanced dynamic wedges, the issue related to overdose to OARs has not been addressed yet [8].

Several researchers have narrated and compared the role of different radiation delivery techniques so that maximal dosimetric benefit and least harm to OARs can be achieved [9,10]. One among them is the Field-in-Field (FIF) technique, also named as forward Intensity Modulated RT (IMRT), which employs multiple small fields of extremely low weights, within the main field, leading to maximizing dose homogeneity to PTV while decreasing the dose to irradiated volume outside the target area. The other planning technique is Volumetric Modulated Arc Therapy (VMAT) in which radiation is delivered through a rotational cone beam with variable shape and intensity as the gantry moves continuously during the treatment along with varying dose rate throughout the arc. 

At Shaukat Khanum Memorial Cancer Hospital and Research Centre (SKMCH&RC), we decided to plan left-sided breast cancer patients, who were booked for radiation therapy after BCS, on two different radiation planning techniques - FIF and VMAT - which are frequently used radiation planning techniques in our center. The purpose of this study was to evaluate dosimetric comparisons between FIF and VMAT radiation techniques with respect to doses to OARs, PTV, homogeneity, and conformity. The volume of data collected during this study will provide us a body of evidence to devise better radiation planning techniques for our breast cancer patients.

## Materials and methods

We retrospectively analyzed 15 left-sided breast cancer patients who underwent BCS. Patients were simulated in head-first supine position and accessories including wing board and headrest were used to maintain a stable position throughout the treatment. Normal free-breathing planning CT scan without contrast was done from chin till diaphragm, with adjacent transverse slices spacing of 5mm. The CT dataset was shifted to the treatment planning system (TPS) (Aria Eclipse, version 15.6; Varian Medical Systems, Palo Alto, USA).

Target and normal tissue delineation

The contours of all the involved OARs including heart, ipsilateral lung, spinal cord, and contralateral breast were outlined by the treating physician as per Radiation Therapy Oncology Group (RTOG) contouring atlas guidelines. The cranial extent of the heart started 5mm below the pulmonary trunk crosses the midline, including the infundibulum of the right ventricle, the right atrium, and the right atrium auricle. The caudal border of the heart was at the level of the diaphragm, included the lowest border of the pericardium and left ventricle. The automated contouring trait of the TPS was used to generate body and lung contours.

The volume of the contralateral breast was defined as the breast tissue encircled by the tangential line between the patient’s midline and the contralateral posterior border was defined as being at the same level as the treated breast. Clinical target volume (CTV) for the breast was delineated by the same radiation oncologist. CTV after breast-conserving therapy (BCT) included all remaining breast tissue and deep fascia excluding muscles and skin. CTV was defined superiorly at the level of second rib insertion, caudally at the level of loss of CT apparent breast tissue, medially sternal rib junction, laterally maxillary line excluding latissimus dorsi muscle, anteriorly skin and posteriorly excluding pectoralis muscle, ribs, and intercostal muscles.

PTV was created by giving the isotropic margin of 10mm to the CTV. Anteriorly, the PTV was corrected for being 5mm inside the skin surface. All OARs, CTV, and PTV were outlined slice by slice on the CT image. After the final approval of all contours, two different radiation treatment plans (VMAT and FIF) were optimized by a medical physicist.

Field-in-field radiotherapy

**Figure 1:**
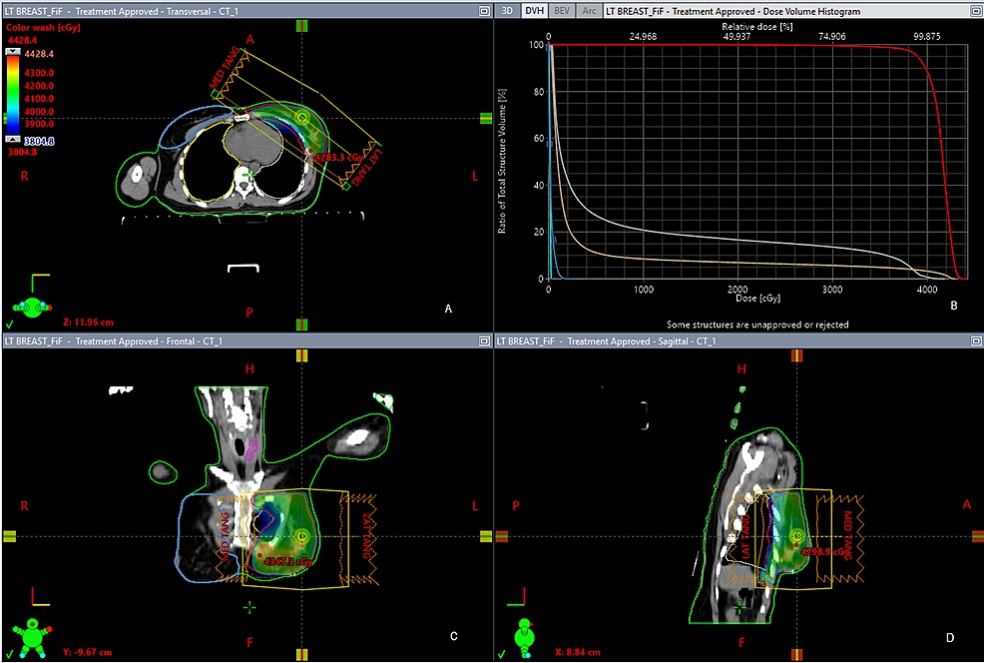
Field-In-Field technique (FIF). A: Transverse view of FIF; B: Dose volume histogram (DVH) showing planning target volume (PTV) (red color), spinal cord (cyan color), contralateral breast (blue color), ipsilateral lung (gray color), heart (pink color); C: Coronal view of FIF; D: Sagittal view of FIF.

In this technique two equally weighted, opposing open tangential photon fields were created against breast PTV, using the same gantry angle as that of the conventional tangential field. In the FIF technique, the gantry angle for the medial tangent was approximately around 300 degrees. Similarly, the gantry angle for the lateral tangent was nearly 120 degrees. The minor variation between gantry angles of patients was according to the patient's tumor location and anatomy. We used five to six segments in one FIF plan, and the number of segments varies from patient to patient. Five to six very low weighted subfields were introduced in the main tangential field in order to decrease the number of hot spots (area of overdose) in the target volume and improve dose homogeneity. All subfields and main fields were merged into one portal. Figure 1 demonstrates PTV coverage and the typical dose distribution of an FIF plan. 

Volumetric modulated arc therapy

**Figure 2:**
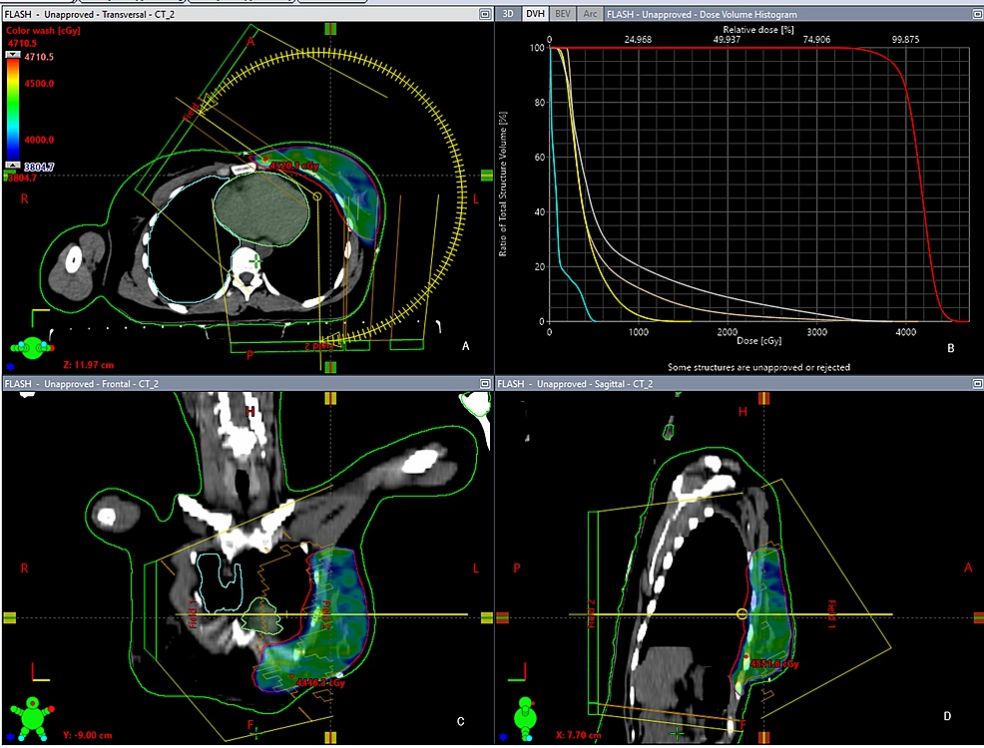
Volumetric Modulated Arc Therapy technique (VMAT); A: Transverse slice of VMAT; B: Dose Volume Histogram (DVH): PTV (red color); Spinal Cord (cyan color); Contralateral Breast (yellow color); Ipsilateral Lung (gray color); Heart (pink color); C: Coronal slice of VMAT; D; Sagittal slice of VMAT.

In order to produce highly conformal dose distribution around breast PTV, two half-arc plans were generated. Within each arc, three parameters of cone-beam were continuously modulated including gantry rotation, dose rate, and multi-leaf collimators (MLCs) so that maximum homogeneity and conformity could be achieved. We achieved a 0.8cm margin between PTV and MLC after using a 1cm virtual bolus on the target volume. Approximately angle of arcs was between 301-179 degrees. In order to avoid secondary dose to normal tissue and hot spot areas, constraints were given to OARs, and target volume and final dose calculations were done. Figure 2 demonstrates PTV coverage and the typical dose distribution of a VMAT plan.

Dosimetric evaluation

In this study, Varian Millennium 120 MLCs (Varian Medical Systems) were used. Patients were planned for a radical dose of 4005cGy in 15 fractions at 267cGy per fraction. Nine patients who were under 50 years were also given an additional boost dose of 1000cGy in five fractions at 200cGy per fraction.

Radiation plans were generated by a senior medical physicist and a review of the radiation treatment plans was done by another senior qualified medical physicist to ensure safe and high-quality care. For the VMAT technique, radiation plans were normalized to 100% target mean dose while for the FIF technique, plans were normalized to field isocenter. The main aim was to cover 95% of the dose to 95% of the volume through both planning techniques. Dose volume histograms (DVHs) were also created for PTV and all OARs including heart, ipsilateral lung, contralateral breast, and spinal cord, and were compared for both radiation planning techniques in all patients. Dose homogeneity index (DHI) and conformity index (CI) were also calculated.

Dose homogeneity index (DHI)

Dose homogeneity index is defined as the ratio between the maximum dose in target volume and the reference isodose. It is calculated from the following formula:

DHI = Imax/RI 

Where Imax = the maximum dose in the target and RI = reference isodose.

Its ideal value is 1 and it increases as the plan becomes less homogenous.

Conformity index (CI)

Conformity index is defined as the ratio between the volume covered by the reference isodose, which according to the International Commission on Radiation Units and Measurements (ICRU) is isodose of 95%, and the target volume, designated as PTV. It is presented by the following equation:

CI = Vri/TV 

Where Vri = volume covered by reference isodose and TV = target volume

The ideal value of CI is 1, as it corresponds to the ideal dose coverage or high conformity. The value of CI greater than 1 indicates that the irradiated volume exceeds the target volume and partly covers the healthy tissue; similarly, its value less than 1 indicates that the target volume is partially radiated.

The PTV doses were analyzed on the basis of the percent volumes receiving at least 95% of the prescribed dose (V95) and the percent volumes receiving 100% of the prescribed dose (V100). Similarly, the maximum point dose of the spinal cord, and the mean doses of the heart and contralateral breast for each plan were also compared. The percentage of the ipsilateral lung volume receiving 12, 16, and 30Gy, and, likewise, the percentage of the heart volume receiving 2 and 10Gy were also evaluated.

Statistical analysis

The Statistical Package for Social Sciences (SPSS Statistics) version 23.0 (IBM Corp., Armonk, USA) was used for statistical analysis. Independent samples t-test was used for comparisons. A p-value of less than 0.05 was considered to be significant.

## Results

The median age of the patients was 41 years. Patient and disease characteristics are displayed in (Table 1).

**Table 1:** Patient and disease characteristics

Characteristic	N (%)
Age in years (Range)	41 (26-58)
Stage I	5 (33)
Stage II	10 (67)
Boost Given	9 (60)
Boost Not Given	6 (40)
Total Dose 5005cGy in 20fr	9 (60)
Total Dose 4005cGy in 15fr	6 (40)

The comparison of plan parameters of FIF technique with VMAT for adjuvant radiation to left-sided breast cancer patients after lumpectomy is outlined in (Table 2).

**Table 2:** Plan parameters with FIF and VMAT techniques PTV 95: Planning target volume receiving at least 95% of the prescribed dose; PTV 100: Planning target volume receiving 100% of the prescribed dose; SD: standard deviation; Gy: Gray; FIF: Field-in-Field; VMAT: Volumetric Modulated Arc Therapy; Heart V10: percentage of heart volume receiving 10Gy; Heart V2: percentage of heart volume receiving 2Gy; Ipsilateral Lung V12: percentage of ipsilateral lung volume receiving 12Gy; Ipsilateral Lung V16: percentage of ipsilateral lung volume receiving 16Gy; Ipsilateral Lung V30: percentage of ipsilateral lung volume receiving 30Gy; DHI: Dose homogeneity index; CI: Conformity index * p<0.05, statistically significant

Parameter	FIF Mean Values	1 SD	VMAT Mean Values	1 SD	P-value*
PTV 95	94.29	3.583	92.14	4.688	0.265
PTV 100	78.29	9.659	68.64	9.410	0.927
Heart Mean Dose(Gy )	3.81	1.800	8.85	3.572	0.024
Heart V10	9.76	4.892	30.76	25.073	0.000
Heart V2	27.54	7.500	97.13	7.887	0.251
Ipsilateral Lung V12	16.73	4.541	33.97	25.923	0.002
Ipsilateral Lung V16	15.00	4.359	19.82	15.253	0.036
Ipsilateral Lung V30	10.73	3.409	4.03	2.789	0.179
Spinal Cord Max Dose	0.18	0.220	7.14	2.722	0.000
Contralateral Breast Mean Dose	0.03	0.062	4.59	1.178	0.000
DHI	1.14	0.068	1.19	0.059	0.037
CI	1.23	0.144	1.00	0.057	0.000

**Figure 3:**
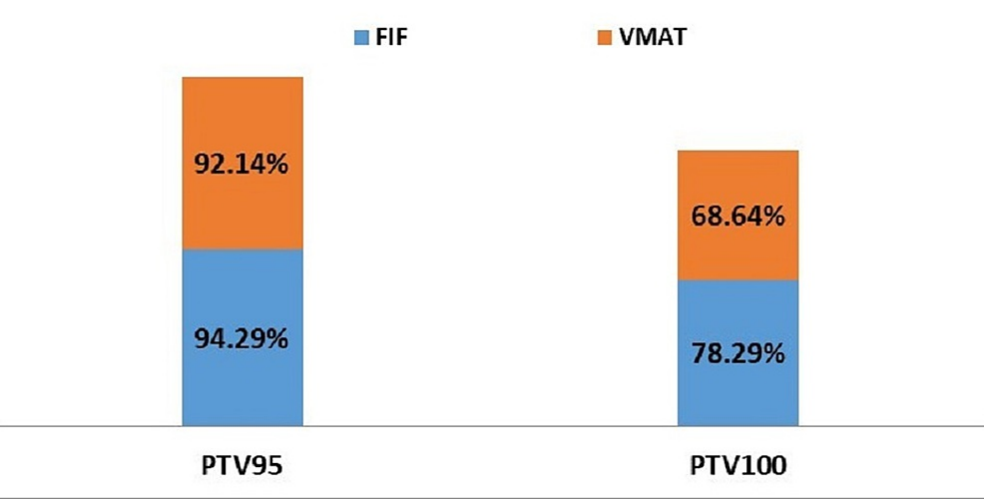
Comparison of percentage target volumes receiving 95% (PTV 95) and 100% (PTV 100) of the prescribed dose by FIF and VMAT techniques FIF: Field-in-Field; VMAT: Volumetric Modulated Arc Therapy

The percentage volumes and standard deviation (SD) of the PTV (left breast) showed that there was no statistically significant difference in terms of the PTV volumes that received 95% of the prescribed dose (p=0.265) and PTV volumes that received 100% of the prescribed dose (p=0.927) when FIF and VMAT techniques were compared (Figure 3).

All patients who were planned on the FIF technique received significantly reduced mean doses to the OARs (heart, spinal cord, contralateral breast, and ipsilateral lung) as opposed to VMAT. When the mean percentage OAR volumes radiated with specified doses were compared between FIF and VMAT, results were in favor of the FIF technique (Table 2).

Heart

The required dose constraints for the heart was the mean heart dose <4Gy. The mean dose of heart achieved by FIF was 3.81Gy versus 8.85Gy by VMAT technique (p=0.024) which is a highly significant difference for left-sided breast cancer patients where it is very difficult to control heart doses. Similarly, the required dose constraints for the percentage heart volume receiving 10Gy and 2Gy were <5% and <30%, respectively. When the mean percentage heart volume receiving 10Gy and 2Gy were evaluated, the FIF technique allowed lower mean percentage values (V10) 9.76% and (V2) 27.54% as compared to the VMAT technique in which (V10) was 30.76% and (V2) 97.13% (p=0.000).

Ipsilateral lung

**Figure 4:**
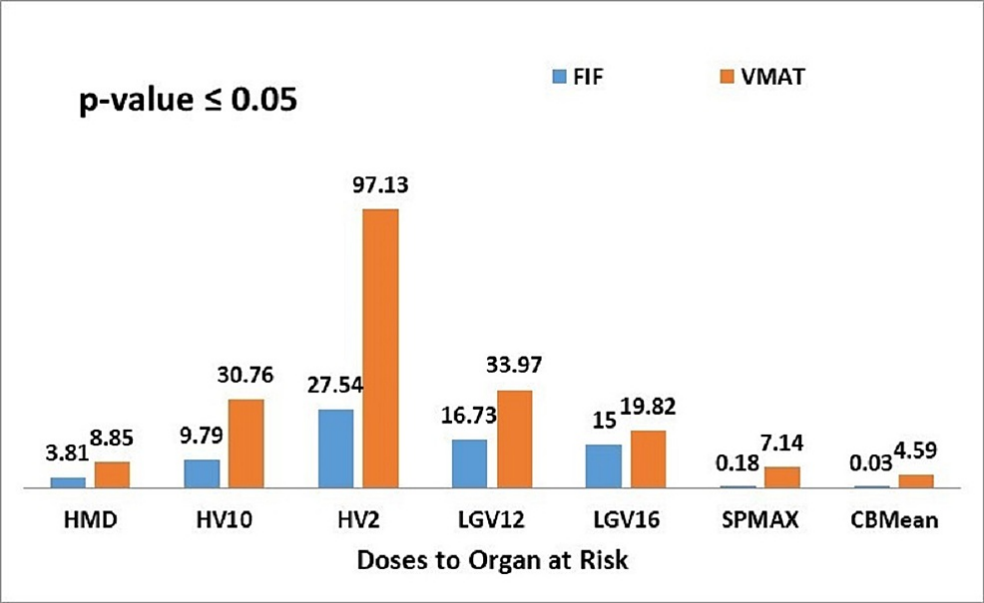
Graphical representation of mean values doses to organs at risk (OARs) by the FIF and VMAT techniques FIF: Field-in-Field; VMAT: Volumetric Modulated Arc Therapy; HMD: heart mean dose; HV10: percentage of heart volume receiving 10Gy; HV2: percentage of heart volume receiving 2Gy; LGV12: percentage of ipsilateral lung volume receiving 12Gy; LGV16: percentage of ipsilateral lung volume receiving 16Gy; SPMAX: Spinal cord maximum dose; CBMean: Contralateral breast mean dose * p<0.05, statistically significant

The required dose constraints for the percentage ipsilateral lung volume receiving 12, 16, and 30Gy were 18%, 20%, and 25%, respectively. When the mean percentage volume of ipsilateral lung receiving 12, 16, and 30Gy was compared between two techniques, the values were significantly reduced with the FIF technique, with a statistically significant p-value for V12 and V16 (p=0.002 for V12 and p=0.036 for V16) (Figure 4). With the FIF technique, we achieved the mean values of V12, V16, and V30 as 16.73%, 15%, and 10.73%, respectively. On the other hand, with the VMAT technique, we got comparatively higher mean values for V12 and V16, i.e., 33.97% and 19.82%, respectively. But V30, that is the percentage mean volume receiving 30Gy, was 10.73% for FIF and 4.03% for VMAT without any statistically significant p-value (p=0.179).

Spinal cord

The maximum spinal cord doses achieved by FIF was 0.18Gy as compared to VMAT, in which it was 7.14Gy (p=0.000), again favoring FIF over VMAT.

Contralateral breast

The mean dose received by the contralateral breast was 0.03Gy by FIF technique and 4.59Gy by VMAT technique with a statistically significant p-value (p=0.000) (Figure 4).

Dose homogeneity and conformity index

**Figure 5:**
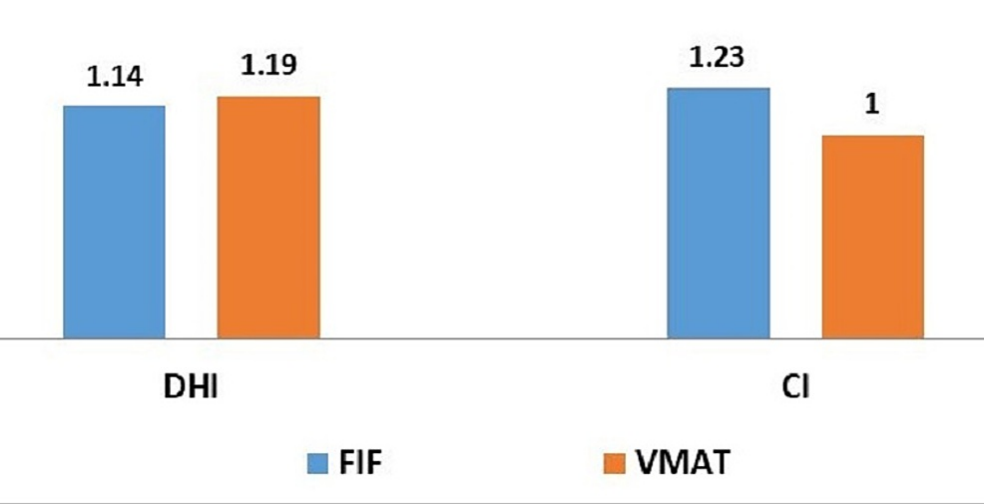
Comparison of dose homogeneity index (DHI) and conformity index (CI) by Field-in-Field (FIF) and Volumetric Modulated Arc Therapy (VMAT) techniques

As the FIF technique offers more homogenous dose distribution when compared to the VMAT technique, the values for the dose homogeneity index are 1.14 and 1.19 respectively with a p-value of 0.03 which is statistically significant. However, the conformity index was much improved with the VMAT technique as compared to FIF and the ideal value of 1 is achieved with VMAT (p=0.000). Therefore, VMAT plans were more conformal as compared to FIF (Figure 5).

## Discussion

In this study, we have evaluated the plan parameters of two different radiation treatment planning techniques in left-sided breast cancer patients treated with breast conservation surgery and have discovered that the FIF technique is superior to VMAT in terms of dose homogeneity and delivering far fewer doses to OARs, specifically the heart. However, conformity is more pronounced with the VMAT technique.

The standard-of-care treatment plan for breast cancer patients includes a tri-modality regimen consisting of systemic agents, surgery, and radiation. Over the period of time, due to advancements in radiation delivering and planning techniques, multiple three-dimensional CT-based whole-breast RT planning techniques have been established with the main aim of delivering fewer doses to OARs and maximizing the homogenous dose distribution in target volume [11,12]. The tangential FIF technique utilizes multiple subfields within the main field; hence, when it is compared to conventional tangential technique with physical wedge filters, it offers a reduction in the number of high-dose regions (hot spots) within the target volume and better control of dose homogeneity [13-15]. The use of MLCs instead of physical wedges in the FIF technique permits reduction of dose-scattering to the OARs and other parts of the body as compared to the conventional tangential wedge field technique [16,17]. Furthermore, the hurdle of contour irregularities and extreme tissue inhomogeneities can also be overcome by adopting the FIF technique [18].

Multiple studies have mentioned the dosimetric comparison of VMAT with 3D conformal radiation techniques [19-21]. When compared to the traditional tangential wedge filters technique, VMAT plans resulted in significantly better tumor coverage and improved homogeneity, but one of the main limitations of the VMAT technique is that a large volume of normal tissues receives low doses [22].

Normal tissue response after radiation delivery depends upon the volume of the tissue irradiated, which will be manifested in the form of organ dysfunction. Malfunctioning organs can be symptomatic or asymptomatic (subclinical) [23]. Multiple Vx values (percentage of organ volume receiving ≥XGy) are related to the biological effect of radiation injury in local tissue. There is usually a strong correlation between individual dosimetric parameters; therefore, any optimal dose threshold is indeterminate to a certain extent. Moreover, it is mandatory for the operator to meticulously compare the similarity of one’s treatment technique with conventional before using any of the limits as a constraint because the existed correlation between dosimetric parameters is dependent on the technique [24]. Similar to the available data in the literature, in this study we have used V2, V10, 12, V16, and V30 values in order to determine the dose constraints for each OAR.

In this study, we found that the FIF technique significantly reduced V12 and V16 values of the ipsilateral lung. Similarly, it is also noticed that the FIF technique remarkably improved the mean contralateral breast dose along with the maximum spinal cord dose when compared to the VMAT technique. Furthermore, heart dose, which is of more concern in left-sided breast cancer patients, is better attained by the FIF technique.

Many published papers in the literature have stated the dosimetric comparison of both conventional and conformal radiation techniques for whole breast radiation [25-27]. De Nile and colleagues reported that PTV coverage was the same using either the VMAT or FIF technique - while the FIF technique gave better results in terms of ipsilateral OAR dose constraints, VMAT was proven suboptimal because the mean doses to OAR were significantly higher [26]. In our study, we have also observed that there is no statistically significant difference in PTV coverage between FIF and VMAT techniques while there is a substantial reduction in radiation doses to normal tissues with the FIF technique. In this study, we do not actually compare the different number of monitor units (MUs) and treatment time between the two techniques, but practically it is observed that FIF takes more time to deliver dose to the patient as compared to VMAT.

The utilization of the FIF technique for whole breast radiation treatment is also advantageous. Haciislamoglu and colleagues compared FIF with VMAT in their study and reported that VMAT offers excellent target coverage and conformity; however, areas of low doses in the contralateral breast and other normal tissues were comparatively greater with VMAT than with FIF. They also concluded that due to lower mean OAR doses achieved through the FIF technique, the chances of long-term side effects are also lower in patients treated by FIF [28]. Our results are compatible with the results of this study.

In our study, we did not include other variables like body mass index (BMI), breast size, and risk of secondary cancers. However, it is a well-known fact that there is a major correlation between breast size and dose homogeneity [29]. Moreover, radiation techniques and long-term side effects like secondary cancers are strongly related. We think that this is the limitation of our study as we have not included these factors.

## Conclusions

In conclusion, our study has demonstrated that the use of the FIF technique for whole breast radiation is more beneficial than VMAT as it offers better results in terms of target coverage, dose homogeneity, and lower doses to OARs. VMAT plan has a slight advantage of improving the CI of the PTV, but it may increase the dose to ipsilateral lung, heart, spinal cord, and contralateral breast. Therefore, the utilization of the VMAT technique for the radiotherapy of the left-sided breast cancer patients is not recommended.
